# The Effect of Delayed Reporting on Mock-Juror Decision-Making in the Era of #MeToo

**DOI:** 10.1177/0886260521997464

**Published:** 2021-02-26

**Authors:** Bailey M. Fraser, Emily Pica, Joanna D. Pozzulo

**Affiliations:** 1 Department of Psychology, Carleton University, Ottawa, Canada; 2 Department of Psychological Science and Counseling, Austin Peay State University, Tennessee USA

**Keywords:** sexual assault, sexual harassment, reporting/disclosure, sexual assault, adult victims, sexual assault

## Abstract

The #MeToo movement has given voice to victims of sexual harassment and assault. In many of these cases, there have been long delays in reporting of the sexual offence (e.g., the Harvey Weinstein case). The purpose of this study was to examine how the type of sexual offence (harassment vs. assault) and the length of delayed reporting (15, 25, 35 years) influenced mock-juror decision-making. Mock-jurors (*N* = 319) read a mock trial transcript depicting an alleged sexual offence and were asked to render a dichotomous verdict, continuous guilt rating, and defendant and victim perception ratings. The data indicated an effect of sexual offence type such that mock-jurors held more favorable perceptions of the defendant when the alleged offence was harassment compared with assault. There also was an effect of delayed reporting such that mock-jurors rendered more guilty verdicts when there was a 25-year delay compared with a 15-year delay. Intriguingly, these results suggest that jurors in sexual offence cases may perceive longer delays in reporting as more believable than shorter delays.

Victims of sexual crimes are often reluctant to report their victimization, often because of feelings of shame and embarrassment, as well as a fear of not being believed (Sable et al., 2010). In addition, victims of sexual crimes often experience physical and mental health issues as a result of their victimization (Potter et al., 2018; Schafran, 1996). The #MeToo movement erupted in October 2017 as an outlet for social media users to come forward with their experiences of sexual harassment and assault (Rotenberg & Cotter, 2018). Following extensive public discourse regarding instances of sexual harassment and assault, another social media movement known as the #TimesUp movement began to take root (Rotenberg & Cotter, 2018). The aim of the #TimesUp movement was to bring forward accusations of sexual harassment or assault against celebrities and prominent figures, such as Harvey Weinstein. Since the wake of the #MeToo movement in 2017 and the #TimesUp movement in 2018, reports of sexual offences have become increasingly widespread (Levy & Mattson, 2020; Rotenberg & Cotter, 2018). For example, researchers have found that within the first six months of the #MeToo movement, police-reported sexual crimes increased by 10% across 30 countries, along with an increase in arrest rates in the United States (Levy & Mattson, 2020). In Canada, there was a 13% increase in reporting of sexual crimes following these movements, compared with the previous year (Rotenberg & Cotter, 2018). Moreover, in October 2017, the month that the #MeToo movement went viral, reports of sexual crimes in Canada were 46% higher than in October of the previous year (Rotenberg & Cotter, 2018).

According to the Canadian Human Rights Commission (CHRC, n.d.), sexual harassment is defined as unwanted verbal or physical behavior, such as unwelcome comments or physical touch. Sexual assault, however, is a more severe form of sexual harassment and is punishable under the Criminal Code of Canada (*Criminal Code*, 1985, c. C-46, s. 271) and the United States Code (Rape and Carnal Knowledge, 10 U.S. Code § 920, Article 120). Many allegations of sexual offending range from sexual harassment to sexual assault. For example, actress Mira Sorvino claimed that in 1995, Harvey Weinstein attempted to massage her shoulders while she was alone with him in her hotel room, while actress Annabella Sciorra claimed that Harvey Weinstein raped her in 1992 (BBC, 2019). Past research has found that the severity of sexual harassment influences mock-jurors’ decisions, such that high severity harassment results in more favorable perceptions of the victim than low severity cases (Cass et al., 2010). However, few studies have examined the effect of sexual offence severity in combination with delayed reporting. Arguably, delayed reporting may have a different influence on mock-jurors’ perceptions and decision-making in sexual harassment versus assault cases. The present study aimed to understand how adult victims’ delayed reporting of instances of sexual harassment and assault influences mock-juror decision-making.

## Delayed Reporting

In many sexual offence cases, the alleged sexual misconduct goes unreported for years. For example, many of the victims in the Harvey Weinstein case reported the sexual offence years, sometimes decades, after the offence occurred (Ransom, 2020). In another high-profile case, Christine Blasey Ford accused Supreme Court nominee, Brett Kavanaugh, of sexually assaulting her 35 years prior while they were both in high school (Abramson, 2018). Although there is a literature examining the effect of delayed reporting on outcomes in historic child sexual abuse (HCSA) cases (e.g., Pozzulo et al., 2010; Read et al., 2006), few studies have examined the impact of delayed reporting in cases with adult victims. This is important to note, as it is possible that delay has a different effect on jurors’ decisions in cases where the victim was an adult versus a child when the crime took place.

Of the few studies that have examined delayed reporting with adult victims, reporting has typically involved short delays (e.g., 0 vs. 18 months; Balogh et al., 2003). Further, Balogh et al. (2003) found that a shorter, immediate delay resulted in higher guilt ratings, more negative ratings of the defendant, and more positive ratings of the victim compared with a longer, 18-month delay. This work suggests that as delay increases, a victim may be perceived as less credible and believable, resulting in fewer guilty verdicts. More research is needed to better understand the impact of delays longer than 18 months.

Although it is possible that the effect of delay differs in cases with child and adult victims, it may still be useful to understand the effect of delay in HCSA cases. Most of the available research on delayed reporting in HCSA cases finds that the effect of delay on juror decision-making is complex. It is typically found that shorter reporting delays are associated with less favorable perceptions of the defendant and higher guilt ratings judged by mock-jurors (e.g., Golding et al., 1999; Pozzulo et al., 2010). However, Bunting (2008) suggests that there is a curvilinear relationship between delayed reporting and outcomes. That is, jurors often favor the victim with both shorter and longer length delays, but favor the defendant with average length delays. This is because with a shorter delay, evidence is likely more readily available and better preserved. With longer delays, the victim is often an adult at the time of reporting, thus they are typically perceived as more credible. In another study, Bunting (2014) found that victims who were adolescents when the abuse occurred were perceived as least credible, whereas adult victims who were younger than 6-years old when the abuse occurred were perceived as most credible. Thus, it is evident that the effect of delay is complex.

Despite these findings, there is limited data regarding how the justice system perceives sexual offence cases in which an adult victim reports the offence years or decades after the crime took place. This is particularly problematic given the rise of sexual offence cases in recent years, notably those cases involving relatively long delays (years or decades in some cases). As delay is likely an influential factor in sexual offence cases and because the effect of delay has been neglected in cases with adult victims, the current study sought to examine the effect of delayed reporting by adult victims on mock-juror decision-making. Further, as sexual offences range from harassment to assault, it is also important to understand how delay influences jurors’ decision depending on the severity of the sexual offence.

## Rape Myths

The criminal justice system responds to sexual offence allegations in two phases. First, the police typically investigate the allegations and then decide whether the case should be prosecuted (Shaw et al., 2017). Then, if sexual offence cases make it to prosecution, juries oftentimes find the defendant ‘not guilty’ (Dinos et al., 2015). It has been suggested that the lack of conviction in sexual offence cases can be partly explained by personally held “rape myths” (Shaw et al., 2017). Rape myths are false beliefs regarding sexual offences, which often shift the blame from the perpetrator to the victim (Burt, 1980; Edwards et al., 2011). For example, one commonly held rape myth is that women secretly like being raped (Edwards et al., 2011). Ultimately, such rape myths can influence juror decision-making in sexual offence cases.

As jurors may hold misconceptions regarding rape, it is important to examine whether the effect of certain factors in sexual offence cases are moderated by mock-jurors’ rape myths. Indeed, previous research has found that mock-jurors’ endorsement of rape myths influences decision-making in sexual offence cases. That is, researchers have found that mock-jurors who hold high acceptance of rape myths are more likely to view the victim’s testimony as less credible and to find the defendant *not* guilty (e.g., Dinos et al., 2015; Hammond et al., 2011; Newcombe et al., 2008; Pica et al., 2017). Given that acceptance of rape myths has been found to be influential in a variety of sexual offence studies, the current study examined whether acceptance of rape myths moderated the effects of sexual offence type and delayed reporting on jurors’ decisions.

## The Current Study

Based on previous research (Cass et al., 2010), we predicted that there would be more guilty verdicts, higher guilt ratings, less favorable perceptions of the defendant, and more favorable perceptions of the victim when the type of alleged sexual offence is described as assault than when the offence is described as harassment. Additionally, based on previous findings (Balogh et al., 2003; Golding et al.,1999; Pozzulo et al., 2010), we predicted that a shorter delay in reporting would result in more guilty verdicts, higher guilt ratings, less favorable perceptions of the defendant, and more favorable perceptions of the victim than when there is a longer delay in reporting. Decade-long delay values were chosen due to the abundance of high-profile, real-life cases in which alleged victims have reported sexual crimes that occurred decades earlier. In addition, decade long delay variables were chosen as previous research has focused on short delays (e.g., Balogh et al., 2003). Moreover, we were interested in understanding whether there were any differences in delay at the higher, extreme end of reporting delays. We also predicted that there would be more guilty verdicts, higher guilt ratings, less favorable perceptions of the defendant, and more favorable perceptions of the victim when there is a shorter delay in reporting and the type of sexual offence is described as assault. That is, the combination of sexual assault and a shorter delay would be more influential than when the sexual offence is described as harassment. Finally, we predicted that participants’ scores on the updated Illinois Rape Myth Acceptance (IRMA) scale would moderate the relationship between the predictor variables and guilt verdicts and ratings, perceptions of the defendant, and perceptions of the victim.

## Method

### Participants

Participants (*N*= 319) were recruited from a University in Eastern Ontario, Canada. Only those who were jury eligible (i.e., Canadian citizen, aged 18-years or older) were permitted to take part in the study. Participants’ ages ranged from 18 years to 53 years (*M*= 20.24, *SD* = 4.80), and the majority of the sample were female (70.5%, *n* = 225). Additionally, the majority of participants classified their ethnicity as White (62.38%, *n* = 199), followed by Black (10.0%, *n* = 32), West Asian (7.5%, *n* = 24), Mixed origin (6.6%, *n* = 21), South Asian (4.4%, *n* = 14), East Asian (4.1%, *n* = 13), Southeast Asian (2.2%, *n* = 7), Latin American (1.9%, *n* = 6), and Indigenous (0.6%, *n* = 2). One participant did not report their ethnicity (0.3%). All participants received course credit for taking part in the study.

### Design

A 2 (type of sexual offence: assault vs. harassment) × 3 (delayed reporting: 15 vs. 25 vs. 35 years) between-subjects factorial design was used.

### Measures

#### Trial transcript.

Six versions of an eight-page mock trial transcript that varied the type of alleged sexual offence (assault, harassment) and the delay in reporting of the sexual offence (15, 25, 35 years) were created (see Online Appendix). The transcript depicted a scenario in which the male defendant was accused of either sexually harassing or assaulting his female coworker while the two were working late at the office. The victim did not report the sexual offence for either 15, 25, or 35 years. All other case details remained constant. Additionally, the type of sexual offence was manipulated by switching the label of the offence (i.e., sexual “assault” vs. sexual “harassment”); however, slight details were changed in some instances (e.g., when describing the event in detail). The following provides an example for how sexual *harassment* was manipulated in the trial transcript:

He came into my office and started making sexual comments about my body and began commenting on how good I looked in my outfit. I ignored him at first, but then he started to ask me questions about my sexual history. I told him that he was being inappropriate and to stop, but he didn’t listen and continued to make sexual comments and ask me sexual questions.

The following provides an example for how sexual *assault* was manipulated in the trial transcript:

He came into my office and started making sexual comments about my body and began commenting on how good I looked in my outfit. I ignored him at first, but then he got really close and began kissing me. I told him that he was being inappropriate and to stop, but he didn’t listen and continued to kiss and grope me. Then he lifted up my dress and began touching me beneath my underwear.

The transcripts began with instructions from the judge, followed by six witness testimonies (i.e., testimony from the victim, the defendant, the police officer who took the victim’s statement, the victim’s friend, the defendant’s friend, and the defendant’s coworker). At the end of the transcript, mock-jurors were provided with closing statements from the Crown and Defence counsel and were provided with instructions from the judge.

#### Verdict form.

Participants were asked to rate the degree to which they believed the defendant was guilty on a 101-point scale (0 = *not guilty*, 100 = *guilty*). Although dichotomous verdicts (i.e., guilty, not guilty) are the only influential decision that real-life jurors reach, it is useful to examine continuous guilt ratings to understand mock-jurors’ subjective feelings of defendant guilt when they are not forced into a dichotomy of guilt. Following ratings of continuous guilt, participants were asked to render a dichotomous verdict (i.e., guilty or not guilty).

#### Victim and defendant ratings.

Participants were asked to rate their perceptions of the victim’s testimony on various dimensions (i.e., honesty, accuracy, reliability, credibility, and believability) on a 101-point scale (0 = *not at all*, 100 = *absolutely*). For example, participants were asked, “how truthful do you believe the alleged victim, Rachel Hamilton, to be” on a scale from 0 [not at all] to 100 [absolutely]. Mock-jurors also were asked to rate the defendant’s testimony in a similar manner. For example, participants were asked, “how credible was Mr. Clement’s [the defendant] testimony” on a scale from 0 [not at all] to 100 [absolutely].

#### Updated IRMA Scale (McMahon & Farmer, 2011).

Participants were asked to complete the 22-item, updated IRMA scale to evaluate endorsement for various rape myths (e.g., “If a girl goes to a room alone with a guy at a party, it is her own fault if she is raped”). Participants rated their agreement with each statement on 1 (strongly agree) to 5 (strongly disagree) Likert scales. Lower scores indicated higher levels of agreement.

#### Manipulation check.

Participants were asked two multiple-choice questions to ensure that mock-jurors understood the trial transcript. The two questions assessed whether mock-jurors remembered information related to the variables of interest. Specifically, participants were asked, “How many years did it take for the victim, Rachel Hamilton, to report the alleged sexual misconduct?” and “Which type of sexual misconduct is Mr. Robert Clement [the defendant] accused of committing?”.

### Procedure

Data were collected entirely online using the survey tool Qualtrics. Participants were given a unique study URL and were then randomly assigned to one of the six conditions. Participants were asked to read the mock trial transcript and then complete a series of questionnaires. Finally, participants completed the manipulation check and were then debriefed and thanked for their participation.

## Results

### Manipulation Check

For data to be included in analyses, it was required that participants answered two of the manipulation questions correctly. One of the questions pertained to the length of delay in reporting of the sexual offence and the other question pertained to the type of alleged sexual offence. There were 379 students who participated in the current study; however, 60 participants were excluded due to incorrectly answering at least one of the questions relating to delayed reporting or sexual offence type. Thus, analyses were based on *N* = 319.

### Dichotomous Verdict

A hierarchical binary logistic regression was conducted to examine the influence of sexual offence type (assault, harassment), delay in reporting (15-year delay was used as the reference group), and the product term representing the two-way interaction on mock-jurors’ dichotomous verdicts (0 = not guilty, 1 = guilty). Block 1 included all main effects. Block 2 included the main effects and the two-way interaction term. Juror characteristics (i.e., gender and race) were initially included as controls in the models; however, these control variables were excluded from the final models as they did not influence the results or improve model fit.^1^1.This is true for the rest of the dependent variables as well. There was a significant effect of delay, such that mock-jurors were 1.8 times more likely to reach a guilty verdict when there was a 25-year delay compared to a 15-year delay,*B*= 0.60,*SE*= 0.28, Wald’s *χ*^2^ (1) = 4.65,*p* = .03, Exp(B) = 1.82, 95% CI [1.06, 3.14]. Interestingly, there was no significant main effect of offence type (Wald’s *χ*^2^ (1) = 2.22, *p* = .14). Additionally, the interaction between offence type and delay was non-significant (Wald’s *χ*^2^ (2) = 0.79, *p* = .67). See Table 1 for the proportion of guilty verdicts rendered in each condition and Table 2 for statistics from the regression models.

**Table 1.** Proportion of Guilty Verdicts (%) Based on Sexual Offence Type and Delayed Reporting.

	Delayed Reporting		
	15 years	25 years	35 years
Sexual offence type			
Assault	42.1	57.1	56.4
Harassment	37.5	52.2	40.8

**Table 2.** Logistic Regression Results for Dichotomous Verdict.

							95% CI for Exp(*B*)	
	*B*	*SE*	Wald	df	*p*	Exp(*B*)	LL	UL
Model 1								
Offence	0.34	0.23	2.22	1	.14	1.40	0.90	2.19
Delay			4.76	2	.09			
25 years	0.60	0.28	4.65	1	.03	1.82	1.06	3.14
35 years	0.37	0.28	1.79	1	.18	1.45	0.84	2.48
Constant	–0.59	0.23	6.71	1	.01	0.56		
Model 2								
Offence	0.19	0.39	0.25	1	.62	1.21	0.57	2.58
Delay			2.34	2	.31			
25 years	0.60	0.40	2.19	1	.14	1.82	0.82	4.01
35 years	0.14	0.40	0.12	1	.73	1.15	0.52	2.52
Offence × delay			0.79	2	.67			
Offence × 25 years	0.01	0.56	0.00	1	.99	1.01	0.34	2.99
Offence × 35 years	0.44	0.55	0.62	1	.43	1.55	0.52	4.57
Constant	–0.51	0.28	3.43	1	.06	0.60		

*Note*. CI = confidence interval; LL = lower limit; UL = upper limit; Offence = type of sexual offence; Delay = delayed reporting.

#### Dichotomous verdict and IRMA.

The updated IRMA (McMahon & Farmer, 2011) scale consists of 22 questions, that examine participants’ support of rape myths. The 22 questions are organized within 4 subscales. Overall, reliability of the scale was 0.92. The first subscale, “She asked for it” (α = 0.81), includes statements such as, “When girls get raped, it’s often because the way they said no was unclear.” The second subscale, “He didn’t mean to” (α = 0.74), includes statements such as, “If a guy is drunk, he might rape someone unintentionally.” The third subscale, “It wasn’t really rape” (α = 0.82), includes statements such as, “If a girl doesn’t physically resist sex—even if protesting verbally—it can’t be considered rape.” The fourth subscale, “She lied” (α = 0.89), includes statements such as, “Rape accusations are often used as a way of getting back at guys.” Lower scores indicate greater agreement with the statement (see Table 3 for means and standard deviations).

**Table 3.** Mock-Jurors’ Means and Standard Deviations for Each of the Updated IRMA Subscales.

	*M*	*SD*
Subscale 1 (*She asked for it*)	4.46	0.65
Subscale 2 (*He didn’t mean to*)	3.94	0.73
Subscale 3 (*It wasn’t really rape*)	4.69	0.58
Subscale 4 (*She lied*)	4.00	0.85

*Note*. IRMA = Illinois Rape Myth Acceptance.

A hierarchical binary logistic regression analysis was conducted to examine whether any of the four IRMA subscales moderated the relationship between the predictor variables (i.e., offence type and delayed reporting) and dichotomous verdict. Model 1 contained the main effects and Model 2 contained the main effects and the two-way interaction terms between the moderator variables (i.e., each of the four IRMA subscales) and the predictor variables (See Table 4 for statistics from the regression models). Importantly, each of the IRMA subscales was mean centered. Model 2 was significant and explained 17.8% of the variance, *χ*^2^ (19) = 41.21,*p* = .002. There was found to be a significant relationship between delayed reporting (15 vs. 25 years) and IRMA subscale 4 (i.e., “She lied”), *B*= –1.22, *SE* = 0.60, Wald’s *χ*^2^ (1) = 4.11, *p* = .04, Exp(B) = 0.29, 95% CI [0.09, 0.96]. Assessment of simple slopes indicated that compared with a 15-year delay, when there was a 25-year delay, mock-jurors’ were more likely to reach a guilty verdict at low levels of IRMA, *B* = .74, *SE* = 0.32, Wald’s *χ*^2^(1) = 5.38, *p* = .02, Exp(B) = 2.10, 95% CI[1.12, 3.94], but not at moderate levels, Wald’s *χ*^2^(1) = 3.21, *p* = .07, or high levels, Wald’s *χ*^2^(1) = 0.12, *p* = .73. There was also found to be a significant relationship between delayed reporting (15 vs. 35 years) and IRMA subscale 3 (i.e., “It wasn’t really rape), *B* = 2.08, *SE* = 1.03, Wald’s *χ*^2^ (1) = 4.08, *p* = .04, Exp(B) = 8.00, 95% CI [1.06, 60.26]; however, assessment of simple slopes revealed no significant differences among the levels of IRMA.

**Table 4.** Logistic Regression Results for IRMA Subscales as Moderators of Dichotomous Verdict.

							95% CI for Exp(*B*)	
	*B*	*SE*	Wald	df	*p*	Exp(*B*)	LL	UL
Model 1								
Offence	0.44	0.25	3.05	1	.08	1.55	0.95	2.52
Delay			4.42	2	.11			
25 years	0.63	0.30	4.28	1	.04	1.87	1.03	3.40
35 years	0.42	0.30	1.87	1	.17	1.52	0.84	2.75
IRMA 1	0.58	0.32	3.32	1	.068	1.79	0.96	3.35
IRMA 2	–0.38	0.22	2.92	1	.088	0.68	0.44	1.06
IRMA 3	–0.54	0.32	2.80	1	.09	0.59	0.31	1.10
IRMA 4	0.57	0.22	6.77	1	.009	1.78	1.15	2.74
Constant	–0.70	0.25	7.74	1	.005	0.50		
Model 2								
Offence	0.31	0.26	1.41	1	.24	1.37	0.82	2.30
Delay			3.53	2	.17			
25 years	0.60	0.32	3.52	1	.06	1.82	0.97	3.41
35 years	0.35	0.32	1.15	1	.29	1.41	0.75	2.66
IRMA 1	0.11	0.68	0.03	1	.87	1.12	0.30	4.21
IRMA 2	–0.43	0.46	0.88	1	.35	0.65	0.27	1.60
IRMA 3	–2.03	0.98	4.29	1	.038	0.13	0.02	0.90
IRMA 4	1.02	0.51	3.40	1	.046	2.78	1.02	7.58
Offence × IRMA 1	–0.05	0.72	0.00	1	.95	0.96	0.23	3.92
Offence × IRMA 2	0.04	0.48	0.01	1	.93	1.04	0.41	2.67
Offence × IRMA 3	0.27	0.85	0.10	1	.75	1.32	0.25	7.00
Offence × IRMA 4	0.60	0.48	1.60	1	.21	1.83	0.72	4.67
Delay [25 years] × IRMA 1	1.26	0.86	2.13	1	.14	3.51	0.65	18.92
Delay [25 years] × IRMA 2	0.30	0.58	0.27	1	.61	1.35	0.44	4.16
Delay [25 years] × IRMA 3	1.00	0.96	1.08	1	.30	2.73	0.41	18.04
Delay [25 years] × IRMA 4	–1.22	0.60	4.11	1	.04	0.29	0.09	0.96
Delay [35 years] × IRMA 1	0.44	0.84	0.27	1	.60	1.55	0.30	8.06
Delay [35 years] × IRMA 2	–0.28	0.58	0.23	1	.63	0.76	0.24	2.36
Delay [35 years] × IRMA 3	2.08	1.03	4.08	1	.04	8.00	1.06	60.26
Delay [35 years] × IRMA 4	–0.63	0.64	0.95	1	.33	0.54	0.15	1.88
Constant	–0.61	0.27	5.01	1	.03	0.55		

*Note*. CI = confidence interval; LL = lower limit; UL = upper limit; Offence = type of sexual offence; Delay = delayed reporting.

### Continuous Guilt

A two-way ANOVA was conducted to examine whether the type of sexual offence (assault, harassment) and delay in reporting influenced mock-jurors’ continuous guilt ratings. Interestingly, there were no significant effects of type of sexual offence (*F*(1, 313) = 1.93, *p* = .17) or delay in reporting (*F*(2, 313) = 0.23, *p* = .80). Additionally, the interaction between offence type and delay was non-significant (*F*(2, 313) = 0.31, *p* = .74). Analyses were conducted to examine whether any of the IRMA subscales moderated the relationship between the predictor variables (i.e., offence type and delayed reporting) and continuous guilt ratings; however, no significance emerged.

### Perceptions of the Defendant

Mock-jurors answered a series of questions regarding their perceptions of the defendant, all of which were significantly correlated (*p* < .01); a composite score was created, such that participant responses were averaged (α = 0.86). Importantly, higher scores indicated more favorable perceptions of the defendant. An ANOVA was conducted to determine whether the type of sexual offence (assault, harassment) and delay in reporting influenced mock-jurors’ perceptions of the defendant. There was a significant effect of offence type, *F*(1, 296) = 4.70, *p*= .03, partial eta^2^ = 0.02. That is, mock-jurors held more favorable perceptions of the defendant when the case involved sexual harassment (*M* = 45.27, *SD* = 21.89) compared to when the case involved sexual assault (*M* = 39.41, *SD* = 23.87). There was no significant effect of delayed reporting (*F*(2, 296) = 0.16, *p*= .85) nor a significant interaction between offence type and delayed reporting (*F*(2, 296) = 0.15, *p*= .86). See Table 5 for means and standard deviations. Analyses were also conducted to examine whether any of the IRMA subscales moderated the relationship between the predictor variables (i.e., offence type and delayed reporting) and mock-jurors’ perceptions of the defendant; however, no significance emerged.

**Table 5.** Mean Ratings (SD) of Mock-Jurors’ Perceptions of Defendant Based on Type of Sexual Offence and Delayed Reporting.

	Delayed Reporting		
	15 years	25 years	35 years
Sexual Offence Type			
Assault	39.78 (22.66)	39.51 (22.00)	38.94 (27.02)
Harassment	46.66 (20.23)	43.20 (22.37)	45.73 (23.48)

### Perceptions of the Victim

Mock-jurors answered a series of questions regarding their perceptions of the victim, all of which were significantly correlated (*p* < .01). Thus, a composite score was created, such that participant responses were averaged (α = 0.97); higher scores indicated more favorable perceptions of the victim. Interestingly, there were no significant effects of type of sexual offence (*F*(1, 313) = 3.23, *p* = .07) or delay in reporting (*F*(2, 313) = 2.16, *p* = .12). Additionally, the interaction between offence type and delay was non-significant (*F*(2, 313) = 0.42, *p* = .66). Analyses were conducted to examine whether any of the IRMA subscales moderated the relationship between the predictor variables (i.e., offence type and delayed reporting) and mock-jurors’ perceptions of the defendant. Model 1 contained the main effects and Model 2 contained the main effects and the two-way interaction terms between the moderator variables (i.e., each of the four IRMA subscales) and the predictor variables. However, Model 2 was non-significant, thus simple slopes were not probed.

## Discussion

The purpose of the current study was to examine how type of sexual offence (i.e., harassment or assault) and delayed reporting of a sexual offence influences mock-jurors’ decisions. Given the abundance of sexual offences reported since the #MeToo movement (e.g., the cases of Harvey Weinstein, Bill Cosby, Larry Nassar, Brett Kavanaugh), it is important to understand how jurors perceive relevant case factors such as delayed reporting and the type of sexual offence in cases involving adult victims. In the Brett Kavanaugh case for example, Christine Blasey Ford accused Kavanaugh of sexually assaulting her 35 years earlier while they were in high school (Abramson, 2018). Despite these allegations, Brett Kavanaugh was later sworn in as a Supreme Court justice (Kim & Wagner, 2018). In the current study, mock-jurors were found to hold less favorable perceptions of the defendant when the type of sexual offence was described as assault compared with harassment; however, only delayed reporting was found to influence mock-jurors’ verdict decisions.

### Delayed Reporting

The results of the current study suggest that delayed reporting has the potential to influence juror decision-making in sexual offence cases involving adult victims. That is, there was an increased likelihood of mock-jurors rendering a guilty verdict when there was a longer delay in reporting (i.e., 25 years) compared with a shorter delay (i.e., 15 years). Interestingly, however, delayed reporting did not influence continuous guilt ratings, perceptions of the defendant, or perceptions of the victim.

The finding that there is an increased likelihood of guilty verdicts with longer delays is somewhat inconsistent with previous findings. That is, in one of the only known studies to examine the effect of delayed reporting with adult sexual assault victims, Balogh et al. (2003) found that a shorter delay (i.e., 0 months) resulted in higher guilt ratings than a longer delay (i.e., 18 months). Despite this apparent inconsistency, it is possible that the current findings align with previous findings but appear inconsistent as only the upper range of delayed reporting effects were captured. Although Balogh et al. (2003) found that longer delays in reporting resulted in lower guilt ratings, it is possible that a delay of longer than 18 months would have resulted in higher guilt ratings. In this case, findings from Balogh et al. (2003) and the current study would provide support for a curvilinear relationship between delayed reporting and guilt ratings, as found in HCSA cases (Bunting, 2008).

An alternative explanation could be that mock-jurors are more likely to reach a guilty verdict when longer delays in reporting coincide with heightened publicity of similar cases. Certain cognitive biases, particularly the availability heuristic (i.e., the tendency to estimate the probability of an event occurring based on the ease at which relevant information is remembered; Tversky & Kahneman, 1973), may increase mock-jurors’ perceived probability of a defendant’s guilt due to the ease at which similar cases come to mind. That is, due to the prevalence of media reportings of sexual offence cases involving longer delays, mock-jurors may find it more believable that a sexual offence occurred when there is a longer delay in reporting. Moreover, mock-jurors may feel that if a victim is reporting an offence decades after an alleged offence occurred, the victim is more likely to be telling the truth.

### Type of Sexual Offence

Sexual offences can include verbal acts of sexual harassment to physical acts of sexual assault (CHRC, n.d.). Instances of sexual harassment and assault can negatively impact a victim’s physical and mental health (Potter et al., 2018; Schafran, 1996). Moreover, sexual offences can have short-term effects such as bruises, broken bones, or contraction of sexually transmitted infections, as well as long-term effects such as post-traumatic stress disorder, insomnia, depression, or anxiety (Potter et al., 2018). Importantly, these short- and long-term effects may also influence other aspects of a victim’s life such as their educational or career goals (Potter et al., 2018). Thus, it is important that the justice system responds fairly to allegations of sexual crimes.

The current study examined the effect of type of sexual offence (i.e., harassment versus assault) on mock-jurors’ decisions. Unexpectedly, type of sexual offence had no influence on verdict, guilt ratings, or perceptions of the victim; however, type of sexual offence was found to influence mock-jurors’ perceptions of the defendant. Specifically, results from the current study found that mock-jurors held more favorable perceptions of the defendant when the type of sexual offence was described as harassment than when it was described as assault. This is not surprising as assault is a more severe form of harassment (CHRC, n.d.) and previous research has found that more severe forms of sexual harassment result in more favorable perceptions of the victim (and arguably less favorable perceptions of the defendant; Cass et al., 2010). Although type of sexual offence influenced mock-jurors’ perceptions of the defendant, it is surprising that offence type did not influence guilt ratings. One explanation is that in the post #MeToo era, sexual harassment and assault may be viewed as more similar in severity.

### Jurors’ Beliefs of Rape Myths

Rape myths refer to a person’s false beliefs regarding a sexual offence, that often result in the blame being shifted from the perpetrator to the victim (Burt, 1980; Edwards et al., 2011). For example, research has found that a commonly held rape myth is that women secretly enjoy being raped (Edwards et al., 2011). Beliefs such as this can be problematic within the criminal justice system as research has found that mock-jurors’ acceptance of rape myths negatively impacts decision-making in sexual offence cases. For example, mock-jurors with high endorsement of rape myths are often more likely to find the defendant not guilty and to rate the victim’s testimony as less credible (e.g., Dinos et al., 2015; Hammond et al., 2011; Newcombe et al., 2008; Pica et al., 2017). As mock-jurors’ acceptance of rape myths has been found to influence decision-making, the current study examined whether acceptance of rape myths moderated the effect of delayed reporting and type of sexual offence on verdict decisions, guilt ratings, and perceptions of both the victim and defendant.

Results from the current study indicate that mock-jurors’ beliefs of rape myths moderated the effect of delayed reporting on verdict decisions. Specifically, compared to a 15-year delay, when there was a 25-year delay mock-jurors were more likely to reach a guilty verdict when they held low acceptance of rape myths, but not moderate or high levels of acceptance; however, this was only found for subscale four (i.e., “She lied”). This is not surprising, as one would expect that other case factors would be less influential with mock-jurors who hold strong acceptance of rape myths. It possible that in the current study, mock-jurors who hold moderate to high levels of rape myth acceptance felt strongly that the victim was lying, and thus were not influenced by delayed reporting.

Despite the current findings, it is important to note that participants in our sample held low levels of rape myth acceptance (see Table 3); thus, it is difficult to conclude that the IRMA subscales do not moderate the relationship between the predictor variables (delayed reporting and type of sexual offence) and mock-jurors’ perceptions and decisions. It is possible that in a sample of participants with high levels of rape myth acceptance, IRMA would moderate the effect of delay and type of sexual offence. For example, it is possible that IRMA may have had a stronger moderating effect had our sample consisted of a greater proportion of men, as research indicates that men often hold higher levels of rape myth acceptance than women (e.g., Bogen et al., 2020; Johnson et al., 1997).

### Limitations and Directions for Future Research

The current study is not without limitations; however, it is important to note that many of the limitations are typical of mock-juror research. First, although participants were jury eligible (i.e., Canadian citizens and 18-years-old or older), the sample consisted of undergraduate students, and thus is not representative of the entire jury-eligible community. In addition, the current study examined individual juror decision-making rather than jury deliberation. Although it can be important to understand jurors’ decisions prior to deliberation, it is also important to understand how case factors influence decisions during deliberation. Future research should examine the effect of delayed reporting in jury deliberation studies. In addition, future research should examine a wider range of delayed reporting. That is, researchers should examine the effect of long delays in reporting that are typical of cases arising from the #MeToo movement (e.g., decades) in comparison with moderate and immediate delays. Finally, as many #MeToo cases involve prominent figures with multiple accusations against them, research should continue to examine relevant variables such as status of the defendant and number of accusations, in combination with delayed reporting.

## Conclusion

This study is one of the first studies to examine the effect of adult victims’ delayed reporting of sexual offences on mock-juror decision-making. Delayed reporting was found to influence mock-jurors’ verdicts (i.e., guilty, not guilty), such that a longer reporting delay (i.e., 25-years) resulted in more guilty verdicts than a shorter delay (i.e., 15-years). Interestingly, in one of the only other studies to examine delayed reporting with adult victims, Balogh et al. (2003) found that shorter delays resulted in more guilty verdicts than longer delays. However, it is possible that with adult victims of sexual offences there is a curvilinear relationship between reporting delay and guilt ratings, similar to what is found in studies involving HCSA cases (Bunting, 2008). Additionally, although type of sexual offence was found to influence mock-jurors’ perceptions of the defendant (i.e., more favorable perceptions of the defendant when the alleged sexual offence is harassment than assault), there was no interaction between delayed reporting and offence type. As there has been a notable increase in reportings of sexual harassment and assault cases following the #MeToo movement (Levy & Mattson, 2020; Rotenberg & Cotter, 2018), more research is needed to understand how delayed reporting influences jurors’ decisions in both sexual harassment and sexual assault cases.

## Supplemental material

Supplemental material for this article is available online. Click here for additional data file. Supplemental Material for The Effect of Delayed Reporting on Mock-Juror Decision-Making in the Era of #MeToo by Bailey M. Fraser, Emily Pica, and Joanna D. Pozzulo, in Journal of Interpersonal Violence

## Author Biographies

**Bailey M. Fraser**, MA, is a PhD student at Carleton University. Her research focuses on eyewitness memory in a developmental context. She also examines factors that influence juror decision-making.

**Emily Pica** is an assistant professor at Austin Peay State University. Her research focuses on wrongful convictions, juror decision-making, and factors that impact eyewitness memory and the collection of eyewitness evidence.

**Joanna D. Pozzulo** is a full professor at Carleton University. Her research focuses on the identification abilities of child and adult eyewitnesses. She also examines factors as they interact with eyewitness identification within a juror decision-making context.
