# Supplementary material for: The Effect of Delayed Reporting on Mock-Juror Decision-Making in the Era of #MeToo
Source: J Interpers Violence. 2021 Feb 26;37(13-14):NP11791–810. doi: 10.1177/0886260521997464 (PMC9253926; doi:10.1177/0886260521997464)
Supplement: Supplemental material for this article is available online. [file sj-pdf-1-JIV-10.1177_0886260521997464.pdf]

Appendix

Trial Transcript

Judge: Robert Clement has been charged with section 271 of the Criminal Code, sexual **assault/harassment**. Under Canadian Law, the Crown has the burden of proving that the defendant is guilty of this charge beyond a reasonable doubt. It is your responsibility to listen to all the evidence presented in this case, to decide the facts, and then to apply the law that I will give to you at the end of this trial. This case will begin with the Crown and Defence presenting their opening statements. These statements are summaries of what will be presented throughout the trial and are not evidence. Each attorney will then present and question witnesses and law enforcement personnel, who will subsequently be cross-examined. Please listen to the following proceedings carefully. Following the testimonies, you will be asked to decide as to whether the defendant, Robert Clement, is guilty or not guilty.

The Crown makes their opening statement.

Crown: The victim, Ms. Hamilton, and the defendant, Mr. Clement, had worked together for four years at an accounting firm. One Winter evening, **15/25 /35** years ago, Mr. Clement acted inappropriately and sexually **assaulted/harassed** Ms. Hamilton while they were both working late at the office. Ms. Hamilton felt ashamed of what had happened, and so up until recently, she kept this sexual **assault/harassment** that occurred **15/25 /35** years ago a secret. Ms. Hamilton says that she finally felt comfortable to come out about the details of the **assault/harassment** and to speak out against Mr. Clement. On that evening, Mr. Clement saw that Ms. Hamilton was vulnerable and so he took advantage of her to get what he wanted. It is therefore, your duty, as representatives of the community, to seek justice by finding Mr. Robert Clement guilty of sexual **assault/harassment**.

The Defence makes their opening statement.

Defence: My client, Mr. Robert Clement, did not sexually **assault/harass** Rachel Hamilton. Yes, it is true that they were around each other when they were both working late at the office. However, the accusations made by the Crown that Mr. Clement sexually **assaulted/harassed** Ms. Hamilton **15/25 /35** years ago are entirely false. It is clear that Ms. Hamilton is making these accusations merely for a monetary settlement and to gain attention from the media. I trust that you will consider all this information accordingly and find my client, Mr. Clement, is not guilty of sexual **assault/harassment**.

The Crown calls their first witness, Nicholas Lowe, and he takes the stand.

Crown: Please state your name and how you know the victim.
Witness: My name is Nicholas Lowe. I am the officer that took Rachel's statement.
Crown: Thank you Mr. Lowe. Can you please explain to me the interaction between you and Rachel when she brought the information of the **assault/harassment** to you?
Witness: Yes, of course. At that time, there multiple high profile sexual misconduct scandals going on in the news involving well-known celebrities. Rachel came into the station saying that these cases impacted her and so she wanted to come forward about her experience. She gave me the name of the person who **assaulted/harassed** her, the defendant, Robert Clement, and

informed me that this happened to her **15/25 /35** years ago while she was working late with Mr. Clement at her accounting job.
Crown: Did you find her story to be credible?
Witness: Yes, I did. Her statement was believable, and the emotions that she expressed while discussing the details of her sexual **assault/harassment** could not have been faked. Also, her account of the **assault/harassment** stayed consistent each time she discussed it.
Crown: How long have you been a police officer Mr. Lowe?
Witness: I have been a police officer for almost 25 years now.
Crown: With your time working as a police officer, do you believe that you would be capable of determining whether someone's story is made-up?
Witness: I'd like to think I could, yes.
Crown: Thank you Mr. Lowe. No further questions.

The Defence cross-examines the witness.

Defence: Mr. Lowe, you claim that you found Rachel Hamilton's statement to be credible, correct?
Witness: Yes, that is correct.
Defence: There was never any doubt that she was telling the truth despite the fact that she waited **15/25 /35 years** to report the alleged sexual **assault/harassment**?
Witness: As I mentioned before, I don't think that someone could have faked the emotions she was showing when she telling me about the **assault/harassment**.
Defence: Do you believe that you are an expert on emotions, Mr. Lowe?
Witness: No. But I believe that I can tell when someone is faking. I have been working with people for almost 25 years now. I have seen a number of victims who have reported cases such as this one.
Defence: So, you are saying that all we have to go on is your *opinion* as to whether Ms. Hamilton was telling the truth?
Witness: I guess that is true, however, Ms. Hamilton told me the story about the **assault/harassment** multiple times and her story has never changed. She has remained consistent.
Defence: Mr. Lowe, how good is your memory for events that have occurred **15/25 /35** years ago?
Witness: Some things are hazy, but I have never experienced a traumatic event like Ms. Hamilton did. I would like to think that a traumatic experience would be fairly easy to remember.
Defence: Exactly, Mr. Lowe, you would like to think that, but you're not certain that it would be easy to remember. No further questions.

The Crown calls their second witness, the victim, Rachel Hamilton, to the stand.

Crown: Can you please state your name for the court?
Witness: My name is Rachel Hamilton.
Crown: Can you tell me your relationship with the defendant, Robert Clement?
Witness: Robert and I were colleagues at an accounting firm I work at**.**
Crown: Thank you. Can you tell me the details of what happened with him the night of the sexual **assault/harassment**?

Witness: Well, we both had to stay late and catch up on some work. It was a busy time and not unusual for one or two people in the office to work late. While I was working in my office he came by and knocked on the door. **He came into my office and started making sexual comments about my body, and began commenting on how good I looked in my outfit. I ignored him at first, but then he started to ask me questions about my sexual history. I told him that he was being inappropriate and to stop, but he didn't listen and continued to make sexual comments and ask me sexual questions. / He came into my office and started making sexual comments about my body, and began commenting on how good I looked in my outfit. I ignored him at first, but then he got really close and began kissing me. I told him that he was being inappropriate and to stop, but he didn't listen and continued to kiss and grope me. Then he lifted up my dress and began touching me beneath my underwear.**
Crown: I'm sorry, Rachel, that must be hard to talk about
Witness: Yes, it is. Thank you.
Crown: Rachel, why did you wait **15/25 /35** years to come forward about the **assault/harassment**?
Witness: I kept telling myself that what had happened was not serious. Once I realized that it was serious, I was worried that no one would believe me.
Crown: So why did you finally decide to come out about the **assault/harassment**?
Witness: I kept hearing about victims of **sexual assault/harassment** coming forward. Listening to their stories made me feel stronger that I could tell my own story. I don't want to let this kind of thing happen to someone else, and now that many victims have been coming forward about sexual **assault/harassment** in the news, I feel more comfortable coming forward myself. I don't want him to damage any more women!
Crown: Are you confident you will get justice against Robert Clement?
Witness: Yes.
Crown: No further questions your Honour.

The Defence cross-examines the witness.

Defence: Rachel, you say this interaction was unwanted correct?
Witness: Yes.
Defence: What did you do when he allegedly **assaulted/harassed** you?
Witness: I asked him to stop.
Defence: Did you think that he understood that you were uncomfortable?
Witness: Well, I asked him to stop. I told him he was being inappropriate but he wouldn't listen.
Defence: Are you sure you are not exaggerating the events of that night in order to get attention?
Witness: No, why would I go publicly about this if it weren't true?
Defence: So, you think no one has ever lied about sexual **assault/harassment**?
Witness: Well no, I am sure that people have, but that is not what is happening. I am telling the truth about that night.
Defence: Were you and Mr. Clement close?
Witness: Well, not really. I mean, I worked with him and I would sit with him in the break room sometimes. We would chat over our lunch break or have lunch together every once in awhile like normal co-workers.
Defence: Why would you do that?

Witness: Because I liked Robert, he was very friendly to me and he was a good co-worker. I was so shocked and upset that he would do this to me.
Defence: Did you continue to speak to him after this alleged **assault/harassment**?
Witness: Well, it was a small office and we work together. I had to talk to him sometimes, but I did try to avoid him when I could.
Defence: There's been many high-profile cases of sexual **assault/harassment** coming forward, with victims getting monetary compensation and media attention, was this a part of your reasoning for coming forward?
Witness: The media did play a role yes, but not in the way you are implying. I wasn't trying to get money from either him or the company.
Defence: Well, please explain your rational then.
Witness: I saw many courageous women coming forward about sexual **assault/harassment**, talking about how they wanted to prevent other women from experiencing what they had to experience.
Defence: So, this has nothing to do with the fact that you could have received some monetary compensation or attention?
Witness: No. Like I said before, I finally felt comfortable enough to come forward, and I don't want him hurting other women around him.
Defence: That is all your Honour. Thank you.

The Crown calls their final witness, Amanda Johnston, to the stand.

Crown: Hi Amanda, can you please state your full name and affiliation with the victim?
Witness: My name is Amanda Johnston. I'm one of Rachel's best friends.
Crown: Amanda, has Rachel disclosed to you the details of the **assault/harassment**?
Witness: Yes, a couple of months ago she told me everything that had happened. I have never seen her that way before. Usually she is so confident and happy. It was terrible seeing her so vulnerable.
Crown: Do you believe Rachel and what she said happened when she was working late with Mr. Robert Clement?
Witness: Yes, absolutely. I know that she would never lie to me.
Crown: How can you be so sure about that?
Witness: We have been friends since grade school; it is easy for me to tell when she is lying.
Crown: Thank you. No further questions.

The Defence cross-examines the witness.

Defence: Amanda, why do you think Rachel waited so long to confide in you about the **assault/harassment**? After all, it was **15/25 /35** years ago that the incident happened.
Witness: Well, she has always been a private person. She doesn't like to talk about things that are bothering her. She doesn't like to burden other people with her problems, and I believe that she knew I would make her go to the police.
Defence: How can you trust her when she kept this from you for **15/25 /35** years?
Witness: I mean, friends don't have to tell each other everything. She didn't want to talk about the **assault/harassment** and I can completely understand that.
Defence: Nothing further your Honour.

The Defence calls their first witness, Laura Christie.

Defence: Good morning. Could you please state your name and your affiliation with the defendant?
Witness: My name is Laura Christie, and I am a close friend of Robert's.
Defence: Mrs. Christie, your friend, Mr. Clement, is being charged with sexual **assault/harassment**. Do you believe that this is something that he would do?
Witness: I would not think so! He's a great friend and a loving father. I could never see him doing something like this.
Defence: Why don't you think Robert could have committed this offence?
Witness: I have been friends with Robert for many years. No one has ever made this type of accusation against him before, and so I just don't think that he did it. I have spent a ton of time with him, in groups of people and alone. **He has never made any comments that are sexual to myself/ never made any sexual advances towards me** or any other women I know. Rachel likely just misunderstood the situation and then later felt embarrassed about it. Robert is a very friendly and considerate person! He is always laughing and talking with everyone. He jokes around a lot. She probably saw that he was a good target.
Defence: Thank you, nothing further your Honour.

The Crown cross-examines the witness.

Crown: Mrs. Christie, Mr. Clement is a close friend of yours, correct?
Witness: Yes, that's correct.
Crown: And do you trust him completely?
Witness: I do.
Crown: Are you familiar with the victim?
Witness: Well, somewhat. I met her a couple of times when I went in to visit Robert while he was working.
Crown: And do you trust her?
Witness: I don't know her enough to trust her. She seemed nice, but I feel that she has made a mistake in this situation. Anyone who knows Robert knows that this is a mistake and he could never do anything like she claims.
Crown: How can you be sure that the alleged **assault/harassment** didn't happen?
Witness: Well, I have known Robert for a long time. This isn't something he'd do. I have never once seen him act like that with anyone. I have been close friends with him for a long time and he has never done anything inappropriate. I trust him completely.
Crown: And so you think this woman is making up this traumatic story?
Witness: I guess so. I feel really badly saying this, and if it weren't Robert I would be more sympathetic to her, but I just know that Robert wouldn't do this.
Crown: Seems like you might not know him quite as well as you thought you knew him. No further questions your Honour.

The Defence calls their second witness, the defendant, Robert Clement, to the stand.

Defence: Please state your name for the Court.
Witness: My name is Robert Clement.
Defence: Please state to the court the nature of your relationship with Rachel Hamilton?
Witness: She was a co-worker of mine at the accounting firm that we worked at.
Defence: Why do you believe that you were accused of this **assault/harassment**?
Witness: I don't know, maybe she wanted some sort of compensation or attention? Those who know me know that I would never have done this. I really can't understand why she would accuse me of this other than to get some attention or money. I have never had any issues at work with my co-workers in the past.
Defence: Can you explain what happened on that night **15/25 /35** year**s** ago?
Witness: Well, I didn't really think twice about it. I hadn't thought about it since the night that it had happened. I was working late that night when I went to her office to ask her a work-related question. Once I entered her office she suddenly and unexpectedly got a little close to me. She began complimenting me and then we ended up kissing. Almost immediately we decided that it wasn't a good idea and so we stopped. That is as far as it ever went.
Defence: Well, if that is all that happened that night, why does Rachel believe that you sexually **assaulted/harassed** her?
Witness: I really have no clue! I don't understand any of this. I would have really hoped that someone would never do this for money or attention, but clearly Rachel is.
Defence: No further questions your Honour.

The Crown cross-examines the witness.

Crown: Hello Mr. Clement.
Witness: Hello.
Crown: You claim you did not sexually **assault/harass** my client **15/25 /35** years ago, correct?
Witness: Yes, that is correct.
Crown: Do you believe that Rachel is making up what happened between you two?
Witness: Well. she is not lying about us having relations. As I said, we had kissed briefly that night, but decided that it was a bad idea and we ended it on good terms. She is making up that I sexually **assaulted/harassed** her.
Crown: Why would she make that up?
Witness: I don't know? She might feel embarrassed about the situation because she was flirting with me and then we decided that it was a bad idea. She is probably angry that nothing more came of our relationship. Maybe she wants attention money or attention? There are so many possibilities. I just know that this accusation is entirely false.
Crown: Or, maybe you are scared of losing your job? Maybe you are embarrassed that it happened so you are denying it? These would be good reasons for you to lie.
Witness: No. I'm not lying about this. I have told the truth.
Crown: That is all your Honour. Thank you.

The Defence calls their final witness, Sophie Arnett, to the stand.

Defence: Hi Sophie how are you today?
Witness: I'm good, thank you.
Defence: Sophie, can you please state your affiliation with the Defendant?

Witness: Robert and I are good friends.
Defence: Where did you meet Mr. Clement?
Witness: We got hired at a firm at around the same time as each other. We worked together for about eight years.
Defence: Mrs. Arnett, how do you feel about the accusations against Mr. Clement today?
Witness: I find them very upsetting. I know Robert would never do anything like this and it makes me so mad that someone could lie like this and destroy the life and career that he has worked so hard for.
Defence: So, you think that it is very unlikely that Robert committed this sexual **assault/harassment**?
Witness: Absolutely! I know what type of person he is! I have seen the way that he treats his co-workers, clients, friends, and family.
Defence: But how can you be so sure about this?
Witness: Like I said, I have seen him as a co-worker, a friend, a family man. I know him very well. He would have no interest in Rachel. He would never have **assaulted/harassed** her. It makes no sense that she would say all of this now, 15/**25 /35** years after she the alleged **assault/harassment** occurred.
Defence: Thank you, Sophie. No further questions your Honour.

The Crown cross-examines the witness.

Crown: Sophie, you say you and Robert worked together in the past, correct?
Witness: Yes, that is correct.
Crown: Can you remember any complaints against Robert from the job that you both worked at?
Witness: Well, yes. There was one co-worker did not like Robert for some reason. She made an official complaint against him. However, she later retracted it and said that it was a misunderstanding and that she forgave him.
Crown: What did the complaint say?
Witness: It said that he was insulting to her and that he did not treat her with respect.
Crown: Insulting women and not treating them with respect? That doesn't really sound like a great man.
Witness: No, it wasn't like that. That woman was equally disrespectful to Robert as he was to her. It was mutual.
Crown: That is all your Honour.

The Crown makes their closing statement.

Crown: Throughout the course of this trial it has become evident that Robert Clement took advantage of Rachel Hamilton by sexually **assaulting/harassing** her **15/25 /35** years ago. Mr. Clement saw the opportunity to sexually **assault/harass** Ms. Hamilton while they were both working late one evening. Ms. Hamilton appeared vulnerable and so Mr. Clement knew that he could take advantage of her. Throughout this trial it has been made clear that Mr. Clement does not highly respect women, and that he will likely continue disrespecting women in the future. Mr. Clement needs to be punished for his behaviour before he does this to anyone else. The way that Mr. Clement behaved on that evening, **15/25 /35** years ago has left my client feeling

vulnerable and emotional ever since. I ask that you trust my client and the evidence presented to you and that you find the defendant, Robert Clement, guilty of sexual **assault/harassment**.

The Defence makes their closing statement.

Defence: My client, Robert Clement, did not sexually **assault/harass** Ms. Hamilton. Throughout the course of this trial there were no hard facts or evidence presented that demonstrate that my client **assaulted/harassed** Ms. Hamilton. Although my client did admit that he and Ms. Hamilton kissed that evening, he has made it very clear that nothing further happened. Mr. Clement has never done anything like this in the past and has no criminal record. Further, this accusation has come up **15/25 /35** years after Ms. Hamilton alleges that the **assault/harassment** occurred. It makes no sense for a victim to wait that long to come forward. The only reason that a victim would come forward after waiting so long is to receive some sort of compensation or attention. I am hopeful that you will all agree that Ms. Hamilton's accusations do not seem credible. I trust that you will examine the evidence and decide that Robert Clement is not guilty of sexual **assault/harassment**.

Instructions to the Jury

Judge: It is your duty to decide whether the Crown has proved Mr. Clement's guilt beyond a reasonable doubt. You have now heard all the evidence that will be called in this case. You must make your decision based on all the evidence presented to you in the courtroom and only on that evidence. You must consider the evidence and make your decision without sympathy, prejudice or fear. You must not be influenced by public opinion. Your duty as a juror is to assess the evidence impartially.

Judge: Mr. Robert Clement is charged with sexual **assault/harassment (**Section 271 of the Criminal Code). You must find Robert Clement not guilty of sexual **assault/harassment** unless the Crown has proved beyond a reasonable doubt that Robert Clement is the person who committed the offence. Specifically, the Crown must prove the following essential element beyond a reasonable doubt:

A person commits **assault/harassment** when, without the consent of another person, he applies force intentionally to that other person, directly or indirectly.

Unless you are satisfied beyond a reasonable doubt that the Crown has proved all this essential element, you must find Mr. Clement not guilty of sexual **assault/harassment**.

If you are satisfied beyond a reasonable doubt of all these essential elements, you must find Mr. Clement guilty of sexual **assault/harassment**.
